# Integrated Transcriptomic and Translatomic Inquiry of the Role of Betaine on Lipid Metabolic Dysregulation Induced by a High-Fat Diet

**DOI:** 10.3389/fnut.2021.751436

**Published:** 2021-10-11

**Authors:** Tengda Huang, Lin Yu, Hongyuan Pan, Zeqiang Ma, Tian Wu, Lifang Zhang, Kang Liu, Qi Qi, Weiwei Miao, Ziyi Song, Haojie Zhang, Lei Zhou, Yixing Li

**Affiliations:** State Key Laboratory for Conservation and Utilization of Subtropical Agro-Bioresources, College of Animal Science and Technology, Guangxi University, Nanning, China

**Keywords:** Ribo-seq, RNA-seq, betaine, translational efficiency, NAFLD

## Abstract

An excessive high-fat/energy diet is a major cause of obesity and linked complications, such as non-alcoholic fatty liver disease (NAFLD). Betaine has been shown to effectively improve hepatic lipid metabolism. However, the mechanistic basis for this improvement is largely unknown. Herein, integration of mRNA sequencing and ribosome footprints profiling (Ribo-seq) was used to investigate the means by which betaine alleviates liver lipid metabolic disorders induced by a high-fat diet. For the transcriptome, gene set enrichment analysis demonstrated betaine to reduce liver steatosis by up-regulation of fatty acid beta oxidation, lipid oxidation, and fatty acid catabolic processes. For the translatome, 574 differentially expressed genes were identified, 17 of which were associated with the NAFLD pathway. By combined analysis of transcriptome and translatome, we found that betaine had the greater effect on NAFLD at the translational level. Further, betaine decreased translational efficiency (TE) for IDI1, CYP51A1, TM7SF2, and APOA4, which are related to lipid biosynthesis. In summary, this study demonstrated betaine alleviating lipid metabolic dysfunction at the translational level. The transcriptome and translatome data integration approach used herein provides for a new understanding of the means by which to treat NAFLD.

## Introduction

With continuous improvement in living standards, dietary changes have significantly increased consumption of high-fat and high-energy foods, resulting in a worldwide increase in the incidence of an overweight and obese population (1). Obesity is a metabolic disease, which increased risk for non-alcoholic fatty liver disease (NAFLD), type 2 diabetes mellitus, cardio-vascular disease, hypertension, coronary heart disease, and cancer (2). NAFLD definition is steatosis of more than 5% of hepatocytes which is not due to drink alcohol (3). The progression of NAFLD steatosis to non-alcoholic steatohepatitis significantly increases the risk of cirrhosis, liver failure, and hepatocellular carcinoma (4). It is estimated that 25% of the world's adults has NAFLD, which is now the number one cause of liver disease in the world (5).

The human liver is an important lipid metabolic organ. It consumes fat through lipid oxidation and secretes fat in the form of very low-density lipoproteins (6). Hepatic triglyceride (TG) accumulation is closely related to NAFLD (7). The pathophysiological mechanism of NAFLD has not been fully elucidated, and there is a lack of effective and specific therapeutic drugs for use in clinical practice (8). Only by properly controlling the metabolic status of patients, through diet and exercise, can treatment be effective (9). Therefore, discovery of new drugs, therapeutic approaches, and therapeutic targets are essential.

Betaine (trimethylglycine) is widely distributed in animals, plants, and microorganisms. Dietary sources include seafood, spinach, and wheat bran (10). Physiologically, betaine functions as a methyl donor and osmotic regulator (11) has been shown to effectively improve NAFLD. In obese mice, betaine attenuated hepatic steatosis by reducing methylation of the microsomal triglyceride transfer protein promoter through elevation of genomic methylation (12). Betaine has also been shown to prevent high-fat diet-induced NAFLD by regulation of the FGF10/AMPK signaling pathway in ApoE^−/−^ mice (13). In mice fed a high-fat diet, betaine prevents the progression of NAFLD, activates AMPK, and decreases acylcarnitine levels (14). Betaine supplementation substantially increased hepatic monoacylglycerol transferase, which is a key enzyme for the synthesis of TG (15). Although genetic, epigenetic, transcriptional, and protein regulatory studies have assessed the role of betaine in NAFLD, little is known of betaine's effect on translational regulation in NAFLD. This lack of knowledge hinders a complete understanding of the betaine mechanisms of role.

Proteins are functional gene products, which are regulated by mRNA generation, degradation, translation, and degradation (16). Translational regulation is indispensable to genetic information transmission, accounting for more than half of all regulatory gene expression (17). A novel method, ribosome footprints (RFPs) sequencing (Ribo-seq) has recently been proposed, providing a genome-wide view of the translational process by deep sequencing of ribosome-protected fragments (RPFs) (18). Ribo-seq enables gene identification and quantification at the genomic translational level, which has been widely used in different species including human (19), mouse (20), zebrafish (21), drosophila (22), rice (23), and maize (24). Ribosome profiling data are characterized by 28–32 nt RFP length, RFP distribution, and translation efficiency (TE) (25), providing valuable information for exploration of biological questions.

In order to investigate the translational effect of betaine on liver TG accumulation, a conjoint technology of RNA-seq and ribosome profiling was used to obtain a snapshot of the translational portrait of mice fed a high fat diet and treated with betaine. Comparisons were made to mice only fed a high fat diet. This study provides a new understanding and a potential means by which to prevent NAFLD.

## Materials and Methods

### Animals

Eight-week-old male C57BL/6 mice were purchased from Guangxi Medical University (Naning, China) and resided in a standard laboratory environment with room temperature of 22 ± 1 °C and 12/12 h light/dark cycle (26). Drinking water is sterilized and accessible.

### Experimental Procedures

**Figure 1:**
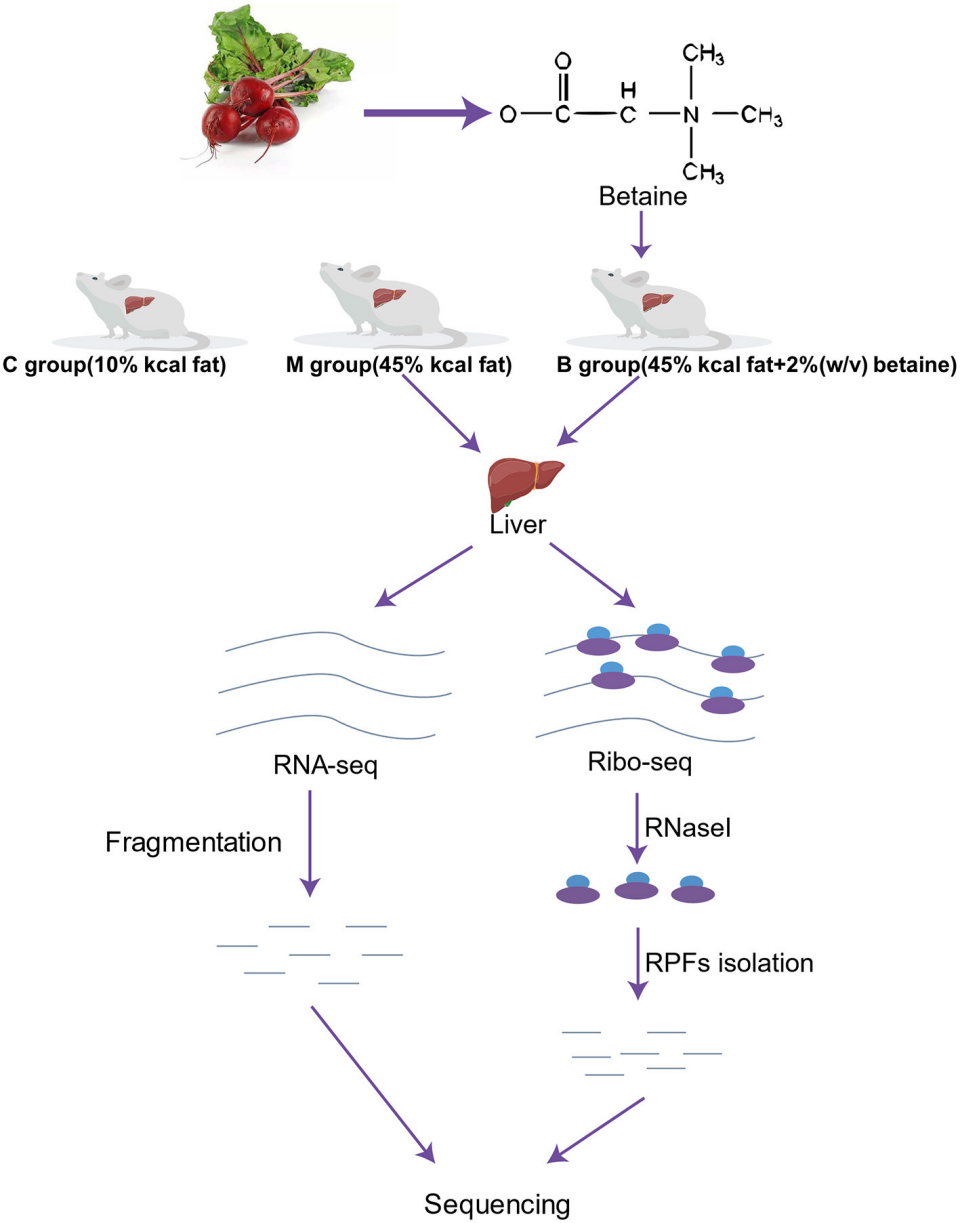
Workflow of the study design and high-throughput sequencing. C, control group; M, model group; B, betaine group.

Figure 1 shows the experimental study design. We randomly selected 18 mice into three groups: a control group (C group, *n* = 6), a high-fat model group (M group, *n* = 6) and a betaine treated group (B group, *n* = 6). The mice in the C group were fed with a standard diet (10% kcal fat). The M and B groups were fed a high-fat diet (45% kcal fat) for 17 weeks (10, 27, 28). Betaine was dissolved in water at a concentration of 2% w/v (29). Betaine was purchased from Beijing Solarbio Science & Technology Co., Ltd., Beijing, China. At the end of the 25th week, the mice were anesthetized for liver collection. The liver samples were frozen in liquid nitrogen and stored at −80°C until analysis. Besides, two mice closest to the average body weight were selected for high-throughput sequencing in group M and group B, respectively.

### Cell Culture

HepG2 cells were purchased from National Collection of Authenticated Cell Cultures (Beijing, China) and cultivated in Dulbecco's modified Eagle's medium (DMEM) supplemented with 10% fetal bovine serum at 5% CO_2_ and 37°C. When there was an 80% confluence, the cells were seeded into 24-well plates. After 48 h, HepG2 cells were divided into two groups: cells in the OAPA group were cultured with oleic acid (OA, 200 mM) and palmitic acid (PA, 100 mM); cells in the OAPA + Betaine group were cultured with oleic acid, palmitic acid, and betaine (2 mM).

### TG Detection and Oil Red O Staining

Liver and cell TG content was detected by a TG assay kit (Applygen Technology, Inc., Beijing, China) in accordance with the manufacturer's instructions. Ten microliters of liver frozen sections were stained with Oil Red O (Servicebio, Wuhan, China). The slides were viewed at 200× and 400× magnification.

### RNA Extraction and Transcriptome Sequencing

Total RNA was isolated from M and B groups using TRIzol® RNA extraction reagent (Ambion, Austin, Texas, USA) (30). The mRNA sequencing libraries were established following the TruSeq^TM^ RNA Sample Preparation Guide (Illumina, San Diego, California, USA) and sequenced with an Illumina HiSeq-2000 for 100 cycles. High quality reads that passed the Illumina quality filters were kept for sequence analysis.

### Extraction of RPFs and Ribo-Seq

RPFs extraction and sequencing were performed by a commercial service company (Chi-Biotech, Shenzhen, China) based on a previous study (31), with minor modification. A total of 90 mg of liver tissue was pre-treated with 100 mg/ml cycloheximide for 15 min, then washed three times by pre-chilled PBS prior to the addition of 2 ml of cell lysis buffer to T-75 flasks. Cell debris was removed by centrifugation at 16,200 × g for 10 min at 4°C. Supernatants were transferred into 1.5 ml pre-cooled tubes and 2 μl of Ribolock RNase Inhibitor (40 U/μl, Fermentas) was added to each tube. RNase I (10 U/μl, Fermentas) was then added at 0.2 μl per tube, followed by incubation for 15 min at 37°C, and the reaction terminated with 1% SDS. The digested samples were layered on the surface of 15 ml of sucrose buffer (30% sucrose). The ribosomes were pelleted by ultracentrifugation at 185,000 × g for 5 h at 4°C. RNA was then extracted using the Trizol method and ribosomal RNA (rRNA) was removed with a Ribo-Zero rRNA Removal Kit (Mouse) (Epicenter). The fragments with the insertion size of ~28 nt were separated and purified from a gel. This fragment was sequenced by Illumina HiSeq-2000 sequencer for 50 cycles.

### Bioinformatic Analysis

For both mRNA and RPF sequencing data sets, clean data were mapped to the reference genome (mm10) through the FANSe2 algorithm (32). The mRNA and RPFs in each sample were normalized by reads per kilo base per million reads (RPKM) (33). Differentially expressed genes (DEGs) were identified with the edgeR package (34). TE was calculated by RPKM _ribosome−profiling_/RPKM _RNA−seq_. Differential TE genes (DTEGs) were calculated by *t*-test. Principal component analysis (PCA), Gene Ontology (GO), Kyoto Encyclopedia of Genes and Genomes (KEGG) analysis, and gene set enrichment analysis (GSEA) were performed using the OmicShare tools, a free online platform for data analysis (http://www.omicshare.com/tools). Protein-protein interaction (PPI) network analysis of DTEGs was conducted using the online STRING website (https://string-db.org/).

### Statistical Analysis

All results were analyzed by GraphPad Prism 8 and expressed as the mean ± standard error. The unpaired two-tailed *T*-test (for two groups) and one-way ANOVA (for multiple groups) were used to identify the significance of difference. If *P* < 0.05, the difference was considered statistically significant.

## Results

### Betaine Significantly Moderated Hepatic Fatty Deposition

**Figure 2:**
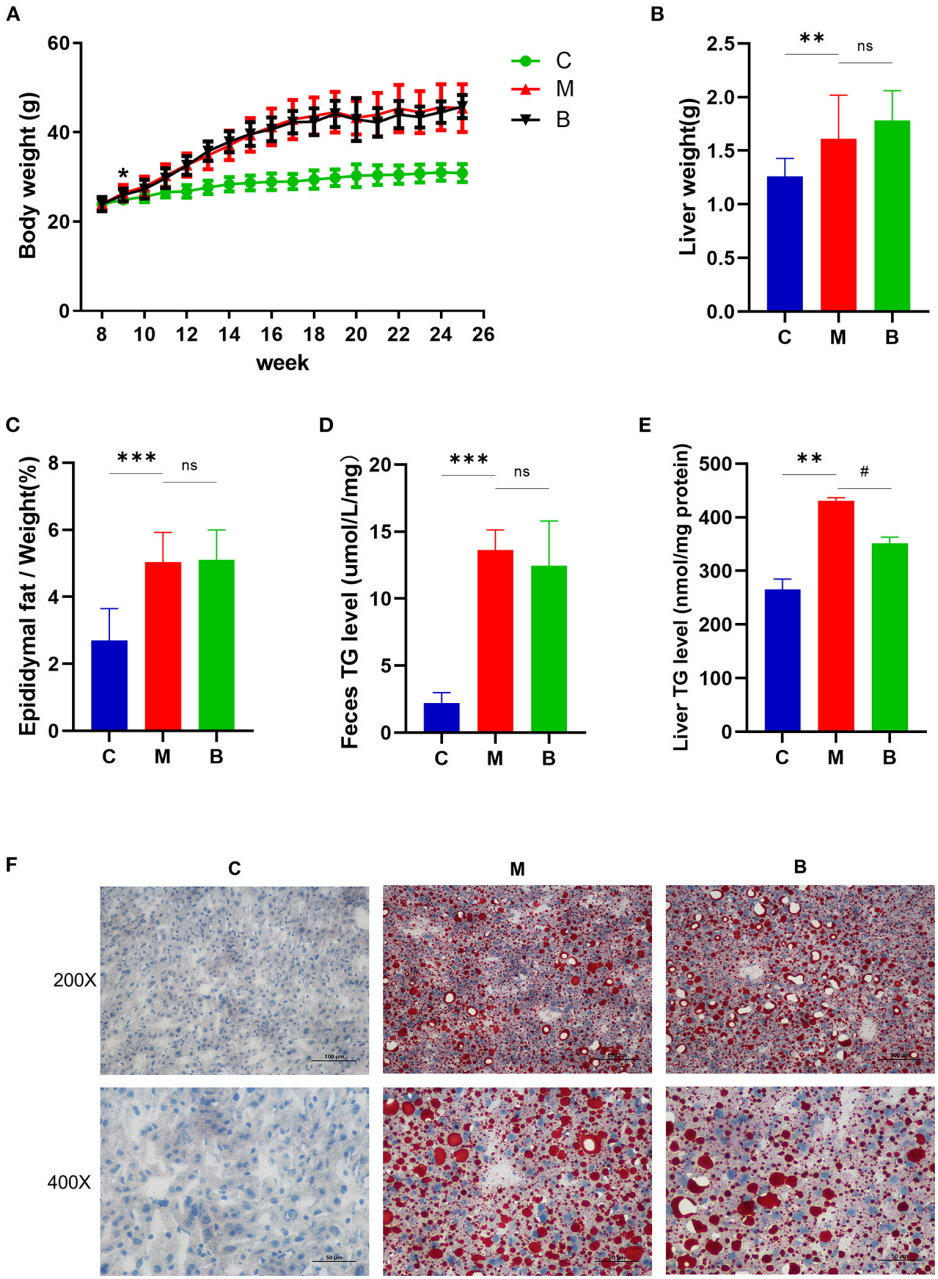
Body weight change and liver lipid levels of C, M, and B group mice. **(A)** The average body weight of mice in three groups at different times. **(B)** Liver weight. **(C)** The epididymal fat/weight (%). **(D)** Feces TG levels. **(E)** Liver TG levels. **(F)** Oil Red O staining (200 × and 400 × magnification) of the liver. *p or ^#^*p* < 0.05; **p < 0.01; ***p < 0.001.

From week 9, the body weight of mice in the M and B groups was significantly higher than that in the C group, indicating successful establishment of the high-fat animal model. There was no significant difference in body weight between the M and B groups (Figure 2A). In terms of liver weight, epididymal fat/body weight ratios, and feces TG levels, significantly higher were found in the M group compared with the C group, with no significant difference between the B and M groups (Figures 2B–D). Compared with the C group, the liver TG levels in the M group increased approximately 1.7 times. There was a significant reduction in hepatic TG levels in the B group compared with the M group (Figure 2E). The regulatory effect of betaine on TG accumulation in cultured HepG2 cells was assessed by adding oleic acid (200 μM, OA) and palmitic acid (100 μM, PA) to DMEM medium to simulate a high lipid environment. Compared with the OAPA group, intracellular TG levels for the OAPA + Betaine group were significantly reduced (Supplementary Figure 1). Moreover, a large number of liver lipid droplets were found in the M group by Oil Red O staining, while the number of lipid droplets was significantly decreased in the B group (Figure 2F). These results demonstrate betaine to reduce high fat diet-induced hepatic fat accumulation in mice.

### Overview of Trancriptome and Translatome

To evaluate betaine regulation of hepatic lipid metabolism, we compared ribosomal profiles of the M and B group livers by Ribo-seq and RNA-seq. At the transcriptional level, 15,847 and 16,215 genes were identified in the M and B groups, respectively (RPKM > 0). At the translation level, 14,308 and 11,063 genes were identified in the M and B groups, respectively (RPKM > 0). The gene expression levels for both the transcriptome and the translatome were similar with normal distribution, log2 RPKM = 3 (Supplementary Figures 2A–D). The identification and quantification information for the transcriptome and translatome are shown in Supplementary Tables 1, 2. PCA of RNA-seq and ribosome profiling showed that biological replicates for the M and B groups to be highly related (Supplementary Figures 2E,F). Supplementary Figure 2G demonstrates the RPFs length distribution peaked at 31 nt in both M and B groups, which was similar to that reported by other species (21–24). The pattern of RPF distribution ratios was similar for the M and B groups (Supplementary Figure 2H). The mRNAs protein-coding sequences (CDS) contained the majority of RPFs in the M and B group, with an average distribution ratio of 59.06 and 64.68%, respectively. The 3′UTR distribution ratio was 37.10 and 29.29%, respectively, for the M and B groups. The 5′UTR distribution ratio was 3.82 and 6.02% for the M and B groups for the RPF distributions, respectively. These data demonstrate the reproducibility of our analysis.

### Differential Transcriptome Analysis

**Figure 3:**
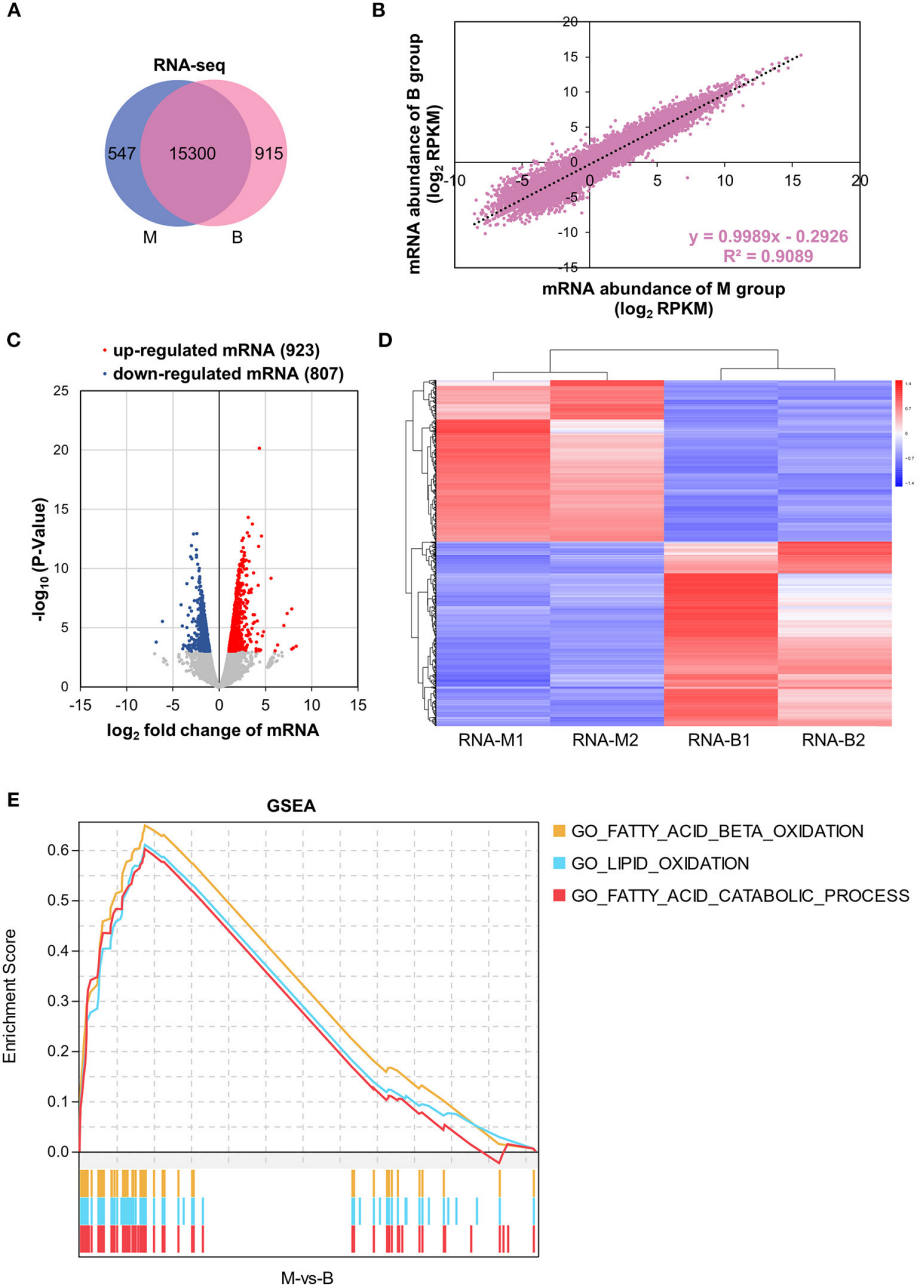
Transcriptome analysis. **(A)** Venn diagram showing the distinct and overlapping genes of the transcriptome. **(B)** Correlation of mRNA abundance. **(C)** Volcano plots of liver DEGs of the transcriptome. **(D)** Heatmap with hierarchical clustering analysis of DEGs of the transcriptome. The color scale indicates the mRNA expression levels; blue is the lowest and red is the highest. **(E)** GSEA of the transcriptome. A pathway of positive Enrichment Score is up-regulated, whereas a pathway of negative Enrichment Score is down-regulated. M1 and M2 represent group M biological repetition 1 and 2. B1 and B2 represent group B biological repetition 1 and 2.

Based on RNA-seq data, transcriptional gene expression was compared between the M and B groups of mice. In total, 15,300 genes were expressed in both the M and B groups, which were highly related (*R*^2^ = 0.9089) (Figures 3A,B). Nine hundred twenty-three up-regulated and 807 down-regulated DEGs of transcriptome were identified based on |log_2_ fold change| > 1 and *P* < 0.001 (Figure 3C). The heat map exhibited 1,730 DEGs based on gene expression level (Figure 3D). In order to further understand the betaine mechanisms of action in hepatic steatosis, GSEA was performed. By GSEA, several positive correlations were identified including; fatty acid beta oxidation, lipid oxidation, and fatty acid catabolic processes (Figure 3E). Supplementary Figure 3 presents the gene set and expression levels of the above up-regulated pathways. These results demonstrated betaine to relieve hepatic steatosis by up-regulation of fatty acid beta oxidation, lipid oxidation, and fatty acid catabolic processes.

### Differential Translatome Analysis

**Figure 4:**
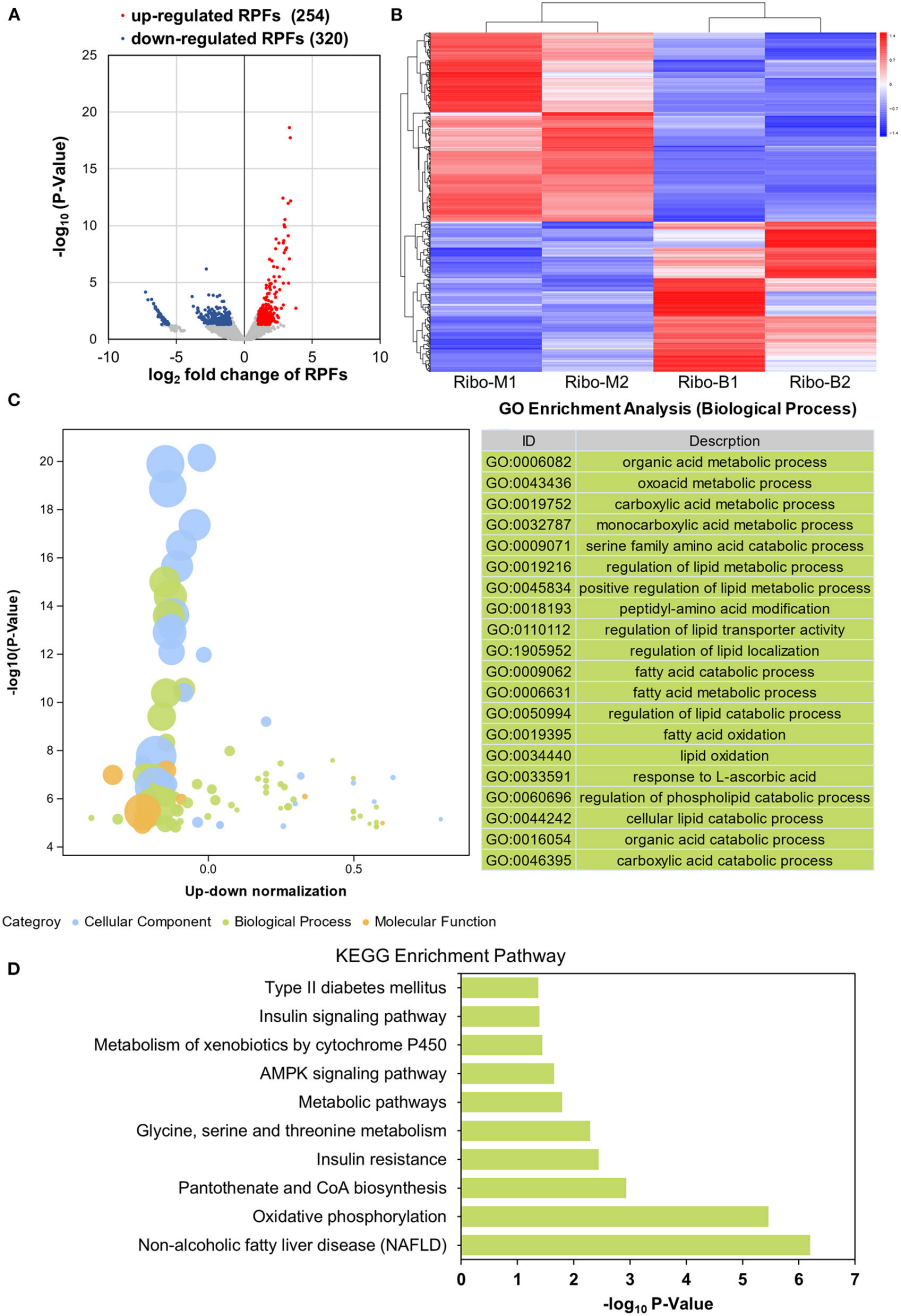
Translatome analysis. **(A)** Volcano plots of liver DEGs of the translatome. **(B)** Heatmap demonstrating the abundance of DEGs. **(C)** An advanced bubble diagram showing GO annotation classification analysis of the DEGs. **(D)** KEGG analysis of DEGs in the translatome.

To determine whether betaine plays a lipid-lowering role at the translational level, the translational expression abundances were assessed. There were 10,813 overlapping genes in the M and B groups, which were highly related (*R*^2^ = 0.7961) (Supplementary Figures 4A,B). A volcano plot was used to screen translatome DEGs by |log_2_ fold change| > 1 and *P* < 0.05. Among those, 254 genes were highly expressed in the B group and 320 genes were highly expressed in the M group (Figure 4A). A heatmap visualized the expression of 574 DEGs (Figure 4B). GO and KEGG enrichment analyses were used to investigate DEGs function. Figure 4C identified the GO biological process terms, classified by –log10 (*P*-value). There was significant enrichment in organic acid metabolic process, oxoacid metabolic process, and carboxylic acid metabolic process. KEGG analysis identified significant enrichment pathways including non-alcoholic fatty liver disease, oxidative phosphorylation, and insulin resistance (Figure 4D). As shown in Supplementary Figure 4C, the KEGG network diagram identified 17 DEGs that Ribo-seq found enriched in the NAFLD pathway. Therefore, betaine can regulate hepatic steatosis at the translational level.

### Joint Analysis of Transcription and Translation

**Figure 5:**
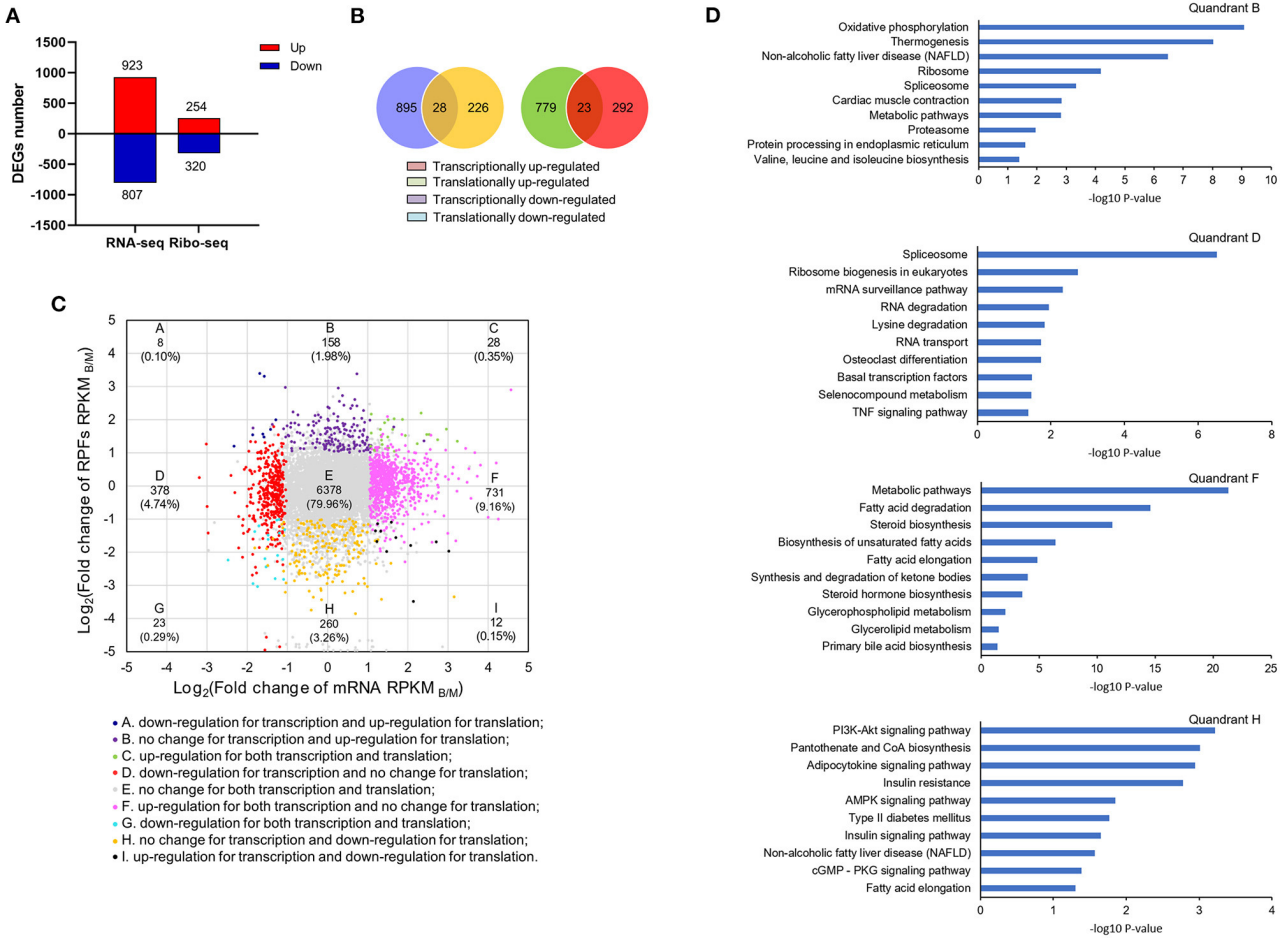
Joint analysis of transcriptome and translatome. **(A)** The number of DEGs at transcriptional and translational levels. **(B)** The relationships of DEGs at transcriptional and translational levels. **(C)** Quadrant diagram of the fold change of the B/M group at transcriptional and translational levels. **(D)** KEGG analysis of genes in classes B, F, D and H.

According to both transcriptome and ribosome profiling data, transcriptional, and translational differences were detected between the M and B groups. There were 923 up-regulated and 807 down-regulated DEGs in the transcriptome, and 254 up-regulated and 320 down-regulated DEGs in the translatome (Figure 5A). Among the DEGs, coordinately DEGs at both the transcriptional and translational levels contained 28 up-regulated and 23 down-regulated DEGs (Figure 5B). Figure 5C shown a quadrant diagram of fold changes in transcriptional and translational differences between the B and M groups. |log_2_(fold change of RPKM) |>1 and *P* < 0.001 for the transcriptome and *P* < 0.05 for the translatome divided the change pattern into nine categories. Class E was comprised of 79.96% of all genes and represented no significant change in transcriptional or translational levels, whereas 0.35 and 0.29% of genes located in class C and G, respectively, had gene changes congruent at the transcriptional and translational levels. Another 19.4% of genes were situated in the six discordantly regulated groups (classes A, B, D, F, H, and I). Supplementary Table 3 lists gene information for the different classes. These results indicated that betaine exerts different regulatory effects at the transcriptional and translational levels.

KEGG enrichment analysis was carried out for classes B, D, F, and H (Figure 5D). Genes in classes B and H were differentially expressed at the translational level without significant difference in transcription. Among them, translationally up-regulated DEGs were significantly associated with pathways of oxidative phosphorylation, thermogenesis, and NAFLD (class B), while translationally down-regulated genes were also observed to be enriched in NAFLD (class H). On the contrary, genes in classes D and F were differentially expressed at transcriptional level without remarkable change at the translational level. In class D, genes with down-regulated expression in transcription were significantly enriched for spliceosome, ribosome biogenesis in eukaryotes, and the mRNA surveillance pathway. There were some transcriptionally up-regulated DEGs (class F), participated in metabolic pathways including, fatty acid degradation, steroid biosynthesis, and biosynthesis of unsaturated fatty acids. By comparison of transcriptome and translatome, we found betaine to more profoundly regulate liver lipid metabolism at the translation level.

### Betaine Significantly Alters TE of mRNAs

**Figure 6:**
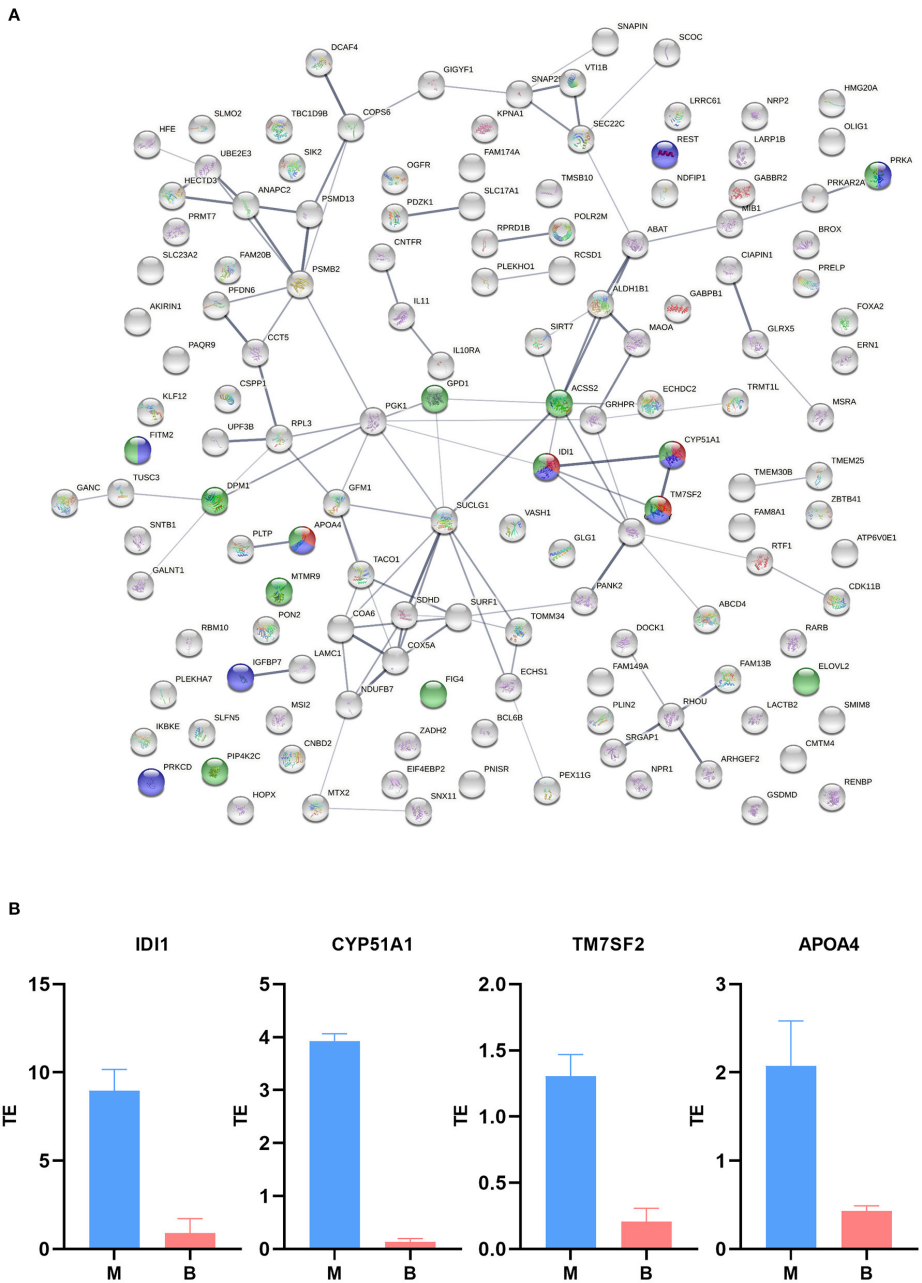
Translation efficiency analysis. **(A)** Interactive and enriched map encoded by DTEGs. Green, lipid biosynthetic processes; purple, regulation of lipid biosynthetic processes; Red, cholesterol biosynthetic processes. **(B)** TE of key functional genes.

TE is a vital translation indicatrix, reflecting the efficiency of mRNA entry into translation. TE is linked to the expression abundance of transcription and translation. Supplementary Figure 5A identified 58 up-regulated DTEGs and 102 down-regulated DTEGs (|log2 fold change of TE| > 2 and *P* < 0.05), demonstrating that gene TE is important for the effects of betaine on hepatic lipid metabolism. Supplementary Figure 5B showed the value of log_2_TE to be mainly distributed between −2 and 3. Functional protein association network analysis was conducted and the interactions of the 160 DTEG coding proteins analyzed to identify target genes of betaine. Results identified 139 nodes and 101 edges that were interconnected, and four DTEGs (IDI1, CYP51A1, TM7SF2, and APOA4) that were enriched in three pathways including lipid biosynthetic processes, regulation of lipid biosynthetic processes, and cholesterol biosynthetic processes (Figure 6A). Compared to the M group, the TEs of IDI1, CYP51A1, TM7SF2, and APOA4 were decreased in the B group (Figure 6B). These results suggest that betaine relieves liver lipid metabolism disorders, possibly through inhibition of gene TE involved in lipid biosynthetic processes, regulation of lipid biosynthetic processes, and cholesterol biosynthetic processes.

## Discussion

NAFLD is a disease with high incidence in the human population, seriously threatening health. There is no definitive treatment for NAFLD. As such, exploration of potential new drugs and possible new treatments is necessary. Herein, *in vivo*, betaine was found to reduce liver fat accumulation induced by a high fat diet (Figure 2E). *In vitro*, betaine significantly reduced OA and PA induced TG accumulation in HepG2 cells (Supplementary Figure 1). This is consistent with the results of previous studies (10).

However, the mechanism of this lipid-lowering effect is largely unknown, especially at the translational level. Based on the ribosome profiling and RNA-seq data, we are not only able to address the genome-wide gene expression changes at the translational level in mice liver with or without betaine treatment but also to investigate the relationship and difference between transcriptional and translational responses. Using Ribo-seq, we identified 254 up-regulated genes and 320 down-regulated genes at the translational level. KEGG analysis showed a significant enrichment in the NAFLD pathway, suggesting that betaine plays an essential role at the level of translation. In order to compare the effects of betaine on transcription and translation, we performed KEGG analysis of DEGs at the single transcriptional level and single translational level. Betaine was found to play a greater translational role in the regulation of NAFLD (Figure 5D). Translational responses contribute to the establishment of complex genetic regulation, which cannot be achieved by controlling transcription alone (35).

Betaine, as a methyl donor, can affect mRNA m^6^A methylation, which can regulate mRNA translation after recognition of m^6^A by reader proteins. For example, YTHDF1 can recognize and bind target mRNA sites modified by m^6^A, enhancing translation initiation factor complex 3 (eIF3) interaction with the ribosomal complex and improving the translational efficiency of the target mRNA (36). Betaine can influence FTO, a demethylase, to regulate lipid metabolism (10). The binding of the YTHDF2 protein to m^6^A sites can inhibit FTO demethylation of the 5′UTR of mRNA, maintaining the m^6^A modification at the 5′UTR region, thus promoting non-classical mRNA translation (37).

Betaine treatment produced 160 DTEGs. Protein–protein interaction enrichment analysis demonstrated betaine reduce hepatic lipid metabolic effects by regulation of lipid biosynthetic and cholesterol biosynthetic processes. Inhibition of TE was mainly due to effects on IDI1, CYP51A1, TM7SF2, and APOA4. IDI1, isopentenyl-diphosphate delta isomerase 1, is a cytoplasmic enzyme involved in the biosynthesis of isoprenoids including cholesterols (38). CYP51A1, cytochrome P450 family 51 subfamily A member 1, is a monooxygenase which catalyzes many reactions involved in drug metabolism and the synthesis of cholesterol, steroids, and other lipids (39). TM7SF2, transmembrane 7 superfamily member 2, catalyzes the reduction of C14-unsaturated sterols during cholesterol biosynthesis from lanosterol (40). APOA4, apolipoprotein A4, enhances insulin secretion, inhibits glucose production in the liver, and increase TG secretion in the liver. Besides, the mRNA and protein of APOA4 in the liver of mice with steatosis induced by high-fat diet was up-regulated by 43 times (41, 42). The imbalance between fatty acid synthesis and decomposition is a key factor leading to fat accumulation in the liver. Betaine can regulate TE of gene participating lipid biosynthesis pathways, thereby reducing hepatic lipid accumulation.

## Conclusions

This study demonstrated betaine to reduce hepatic lipid metabolic disruption, which provides a feasible direction for improvement and treatment of NAFLD. Besides, betaine can reduce the TE of genes related to lipid synthesis and increase fatty acid beta oxidation, lipid oxidation, and fatty acid catabolic processes. Our joint analysis based on transcriptome and translatome provides a new way to narrow the range of key functional genes. These provide a novel comprehension of betaine and liver lipid metabolism.

## Data Availability Statement

The datasets presented in this study can be found in online repositories. The names of the repository/repositories and accession number(s) can be found here: https://www.ncbi.nlm.nih.gov/, GSE181077.

## Ethics Statement

The animal study was reviewed and approved by Animal Protection and Utilization Committee of Guangxi University (No. GXU2019-063).

## Author Contributions

TH, LY, LZho, and YL conceived the project and design the protocol. HP, ZM, LZha, KL, TW, QQ, and WM performed the experiments. TH, ZS, and HZ performed the data analysis. TH, LZho, and YL wrote the manuscript. All authors contributed to the article and approved the submitted version.

## Funding

This work was supported by grants from the National Key R&D Program of China (2018YFD0500402), Guangxi Science Foundation for Distinguished Young Scholars (2020GXNSFFA297008), Guangxi Science and Technology Base and Talents Project (AD18281085), Guangxi Natural Science Foundation (2019GXNSFDA245029), Guangxi Hundred-Talent Program, State Key Laboratory for Conservation and Utilization of Subtropical Agro-bioresources (SKLCUSA-a202006), and Training Project of High-level Professional and Technical Talents of Guangxi University.

## Conflict of Interest

The authors declare that the research was conducted in the absence of any commercial or financial relationships that could be construed as a potential conflict of interest.

## Publisher's Note

All claims expressed in this article are solely those of the authors and do not necessarily represent those of their affiliated organizations, or those of the publisher, the editors and the reviewers. Any product that may be evaluated in this article, or claim that may be made by its manufacturer, is not guaranteed or endorsed by the publisher.

## Supplementary Material

Supplementary Figure 1 HepG2 cellular TG levels.

Supplementary Figure 2 Overview of transcriptome and translatome. **(A–D)** Distribution of the mRNA and RPFs abundance in M and B groups. **(E,F)** PCA of RNA-seq and Ribo-seq. **(G)** Length distribution of RPFs. **(H)** The percentage of RPFs located in the CDS, 5′UTR, and 3′UTR.

Supplementary Figure 3 Gene expression heatmap of the three up-regulated pathways.

Supplementary Figure 4 Ribo-seq analysis. **(A)** Venn diagram showing the distinct and overlapping genes of the translatome. **(B)** Correlation of RPF abundance. **(C)** NAFLD pathway. Red stands for up-regulated DEGs and green stands for down-regulated DEGs.

Supplementary Figure 5 DTEGs analysis. **(A)** Volcano plots of DTEGs. **(B)** TE distribution.
