# Supplementary figures and images for: Integrated Transcriptomic and Translatomic Inquiry of the Role of Betaine on Lipid Metabolic Dysregulation Induced by a High-Fat Diet
Source: Front Nutr. 2021 Oct 11;8:751436. doi: 10.3389/fnut.2021.751436 (PMC8542779; doi:10.3389/fnut.2021.751436)

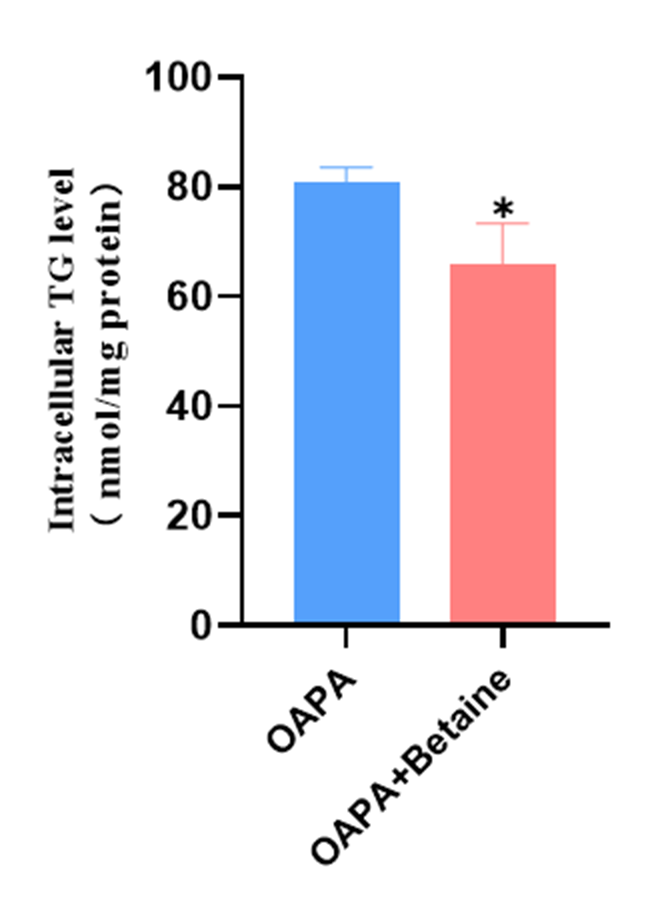

Supplement: Supplementary Figure 1 — HepG2 cellular TG levels. [file Image_1.TIF]

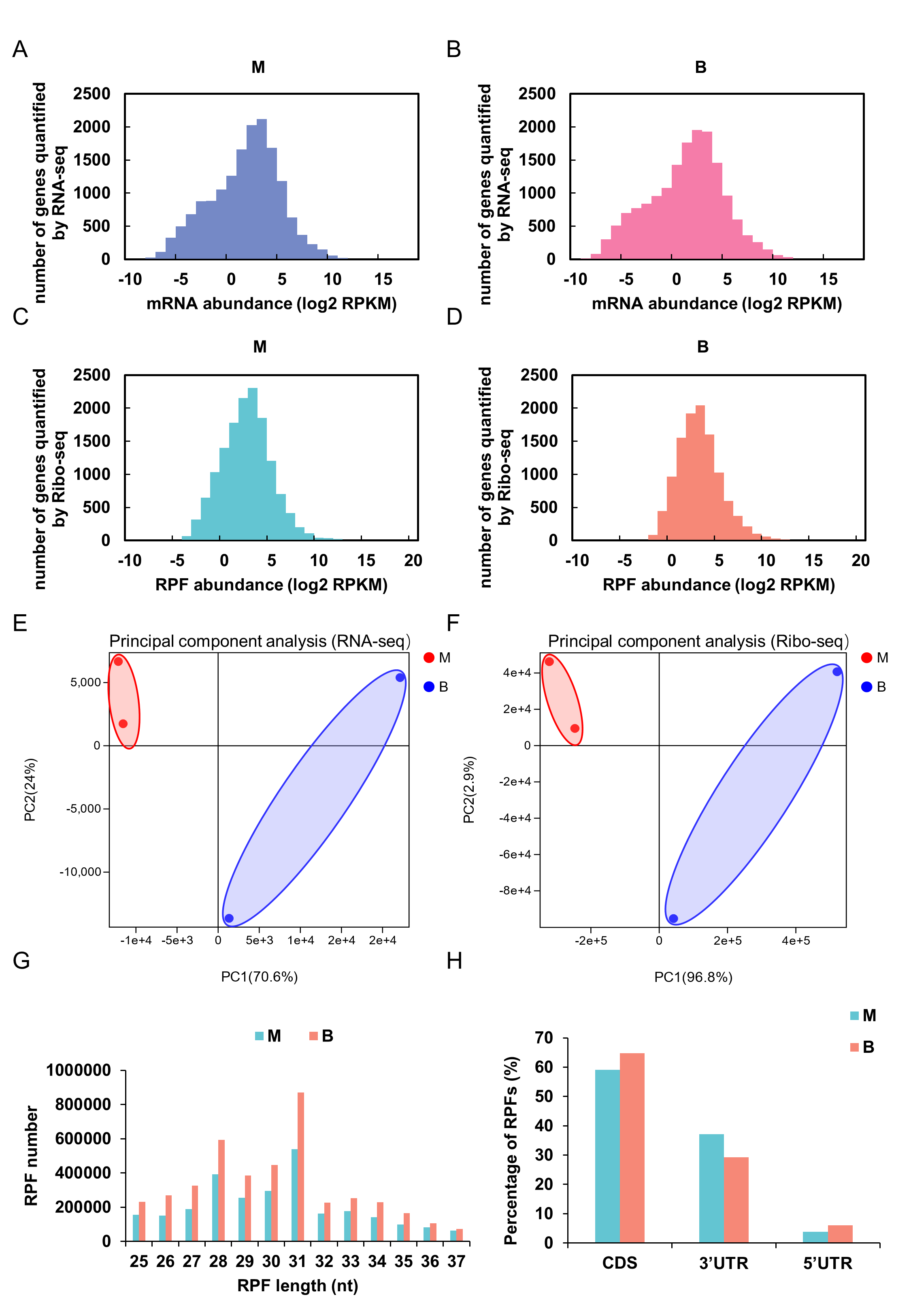

Supplement: Supplementary Figure 2 — Overview of transcriptome and translatome. (A–D) Distribution of the mRNA and RPFs abundance in M and B groups. (E,F) PCA of RNA-seq and Ribo-seq. (G) Length distribution of RPFs. (H) The percentage of RPFs located in the CDS, 5′UTR, and 3′UTR. [file Image_2.TIF]

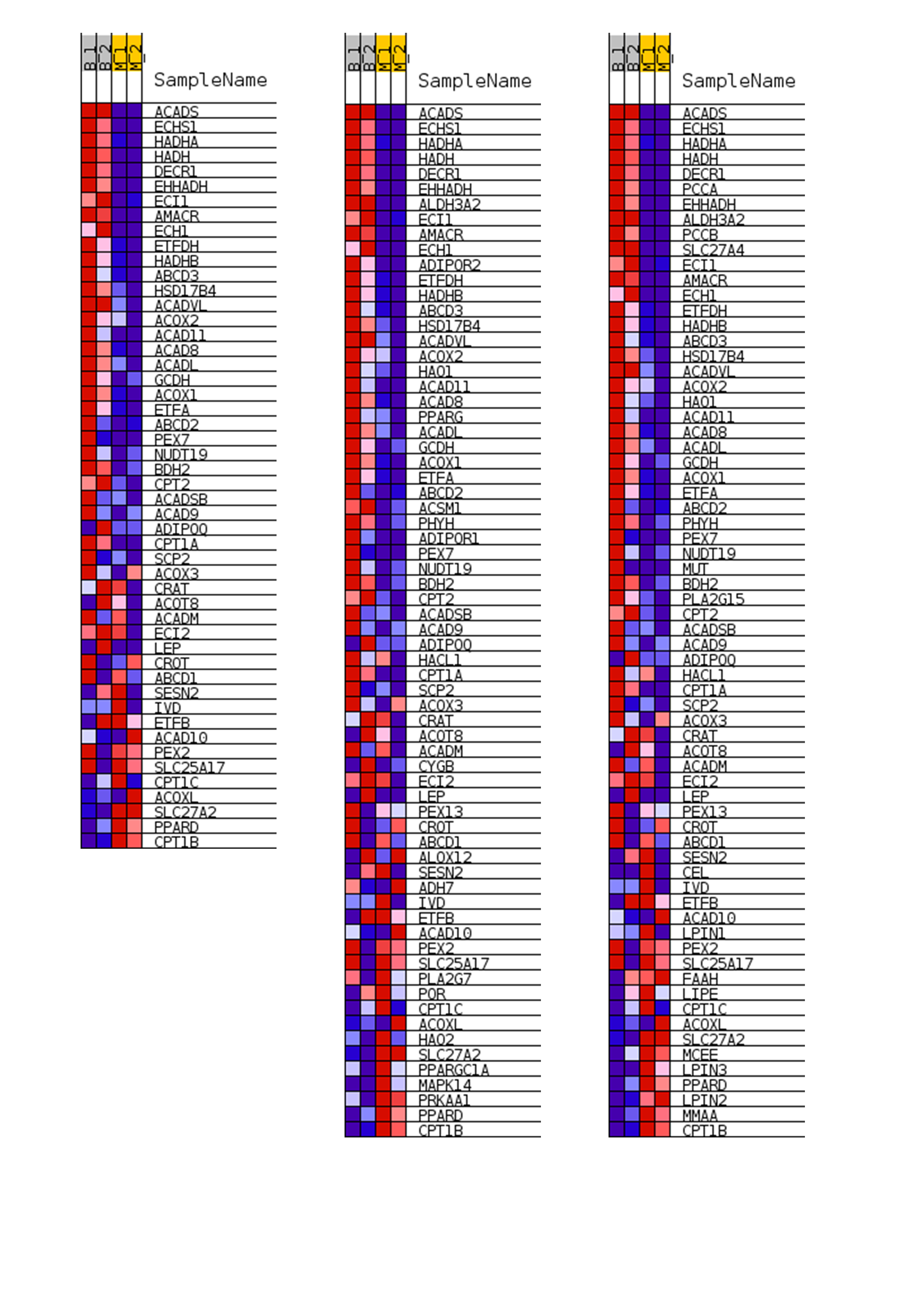

Supplement: Supplementary Figure 3 — Gene expression heatmap of the three up-regulated pathways. [file Image_3.TIF]

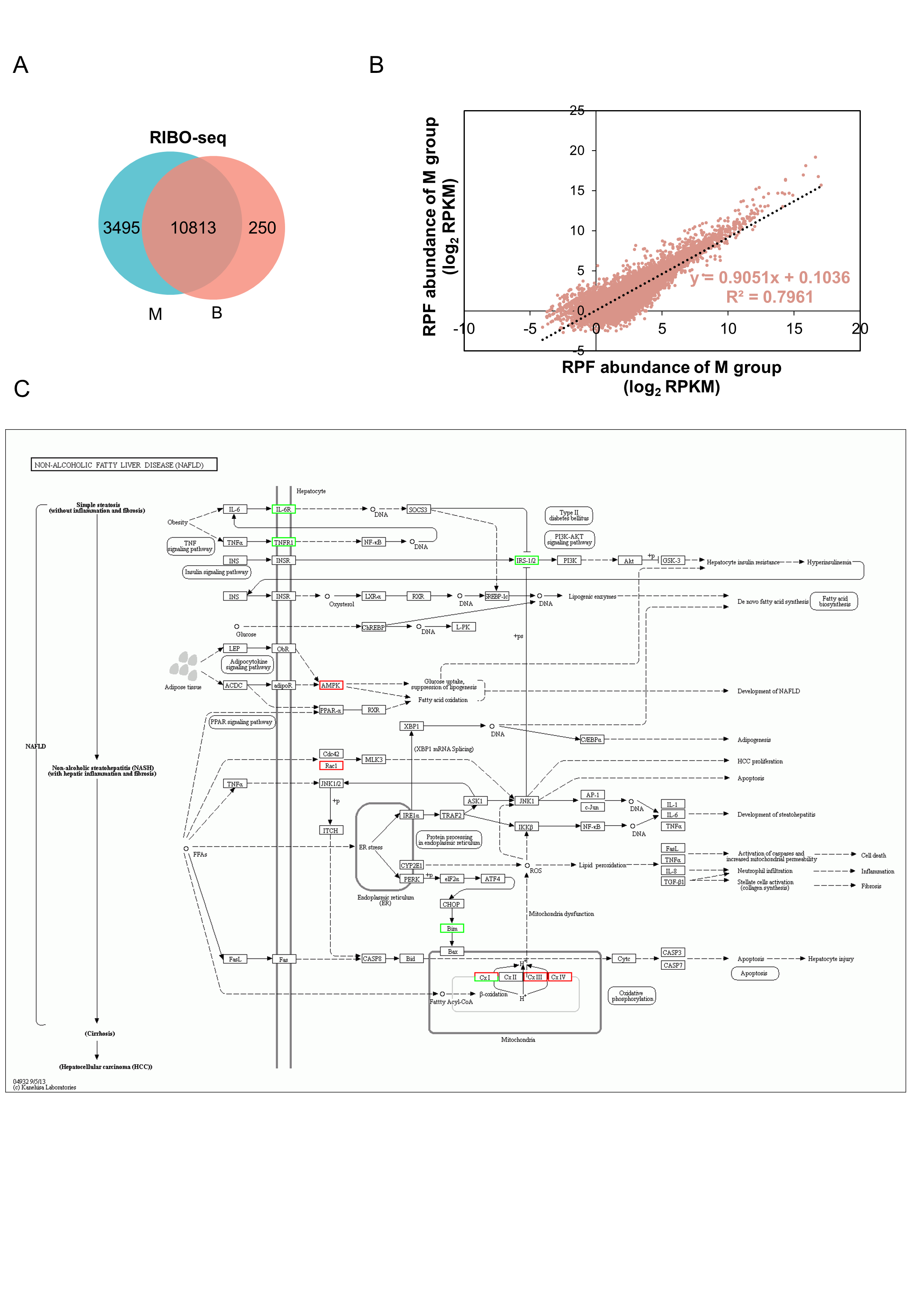

Supplement: Supplementary Figure 4 — Ribo-seq analysis. (A) Venn diagram showing the distinct and overlapping genes of the translatome. (B) Correlation of RPF abundance. (C) NAFLD pathway. Red stands for up-regulated DEGs and green stands for down-regulated DEGs. [file Image_4.TIF]

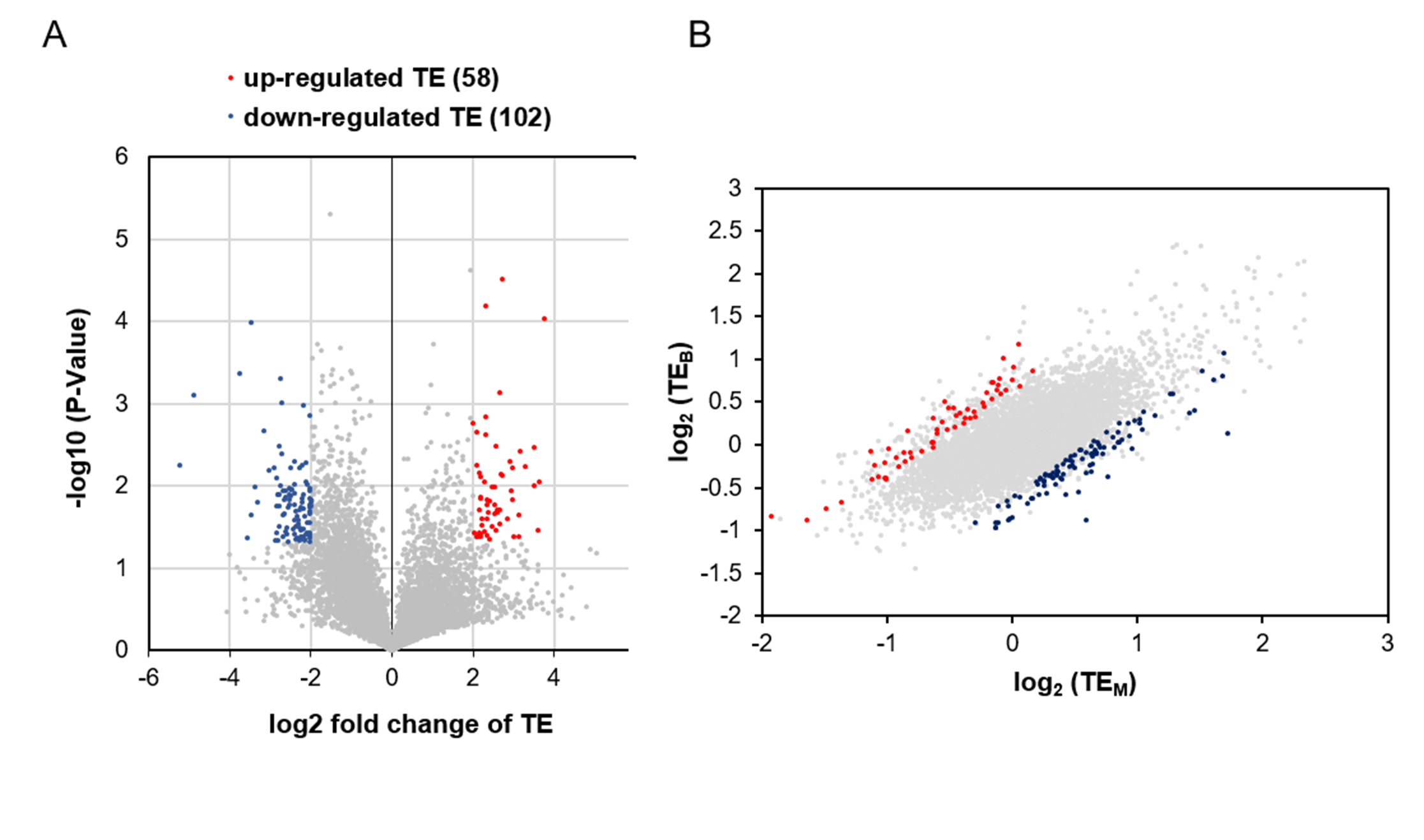

Supplement: Supplementary Figure 5 — DTEGs analysis. (A) Volcano plots of DTEGs. (B) TE distribution. [file Image_5.TIF]
